# Feasibility and safety of cangrelor in patients with suboptimal P2Y_12_ inhibition undergoing percutaneous coronary intervention: rationale of the Dutch Cangrelor Registry

**DOI:** 10.1186/s12872-021-02093-4

**Published:** 2021-06-12

**Authors:** A. Selvarajah, A. H. Tavenier, W. L. Bor, V. Houben, S. Rasoul, E. Kaplan, K. Teeuwen, S. H. Hofma, E. Lipsic, G. Amoroso, M. A. H. van Leeuwen, J. M. ten Berg, A. W. J. van ‘t Hof, R. S. Hermanides

**Affiliations:** 1 Department of Cardiology, Isala Hospital, Zwolle, The Netherlands; 2 Department of Cardiology, St. Antonius Hospital, Nieuwegein, The Netherlands; 3 Department of Cardiology, Zuyderland Medical Center, Heerlen, The Netherlands; 4 Department of Cardiology, Venlo VieCuri Medical Center, Venlo, The Netherlands; 5 Department of Cardiology, Catharina Hospital, Eindhoven, The Netherlands; 6 Department of Cardiology, Medical Center Leeuwarden, Leeuwarden, The Netherlands; 7 Department of Cardiology, University Medical Center Groningen, Groningen, The Netherlands; 8 Department of Cardiology, OLVG Hospital, Amsterdam, The Netherlands; 9 Cardiovascular Research Institute Maastricht (CARIM), Maastricht, The Netherlands

**Keywords:** Cangrelor, P2Y_12_ inhibitors, Percutaneous coronary intervention, Platelet inhibition

## Abstract

**Background:**

Despite the advances of potent oral P2Y_12_ inhibitors, their onset of action is delayed, which might have a negative impact on clinical outcome in patients undergoing percutaneous coronary intervention (PCI). Trials conducted in the United States of America have identified cangrelor as a potent and rapid-acting intravenous P2Y_12_ inhibitor, which has the potential of reducing ischemic events in these patients without an increase in the bleeding. As cangrelor is rarely used in The Netherlands, we conducted a nationwide registry to provide an insight into the use of cangrelor in the management of patients with suboptimal platelet inhibition undergoing (primary) PCI (the Dutch Cangrelor Registry).

**Study design:**

The Cangrelor Registry is a prospective, observational, multicenter, single-arm registry with cangrelor administered pre-PCI in: (1) P2Y_12_ naive patients with ad-hoc PCI, (2) patients with STEMI/NSTEMI with suboptimal P2Y_12_ inhibition including (3) stable resuscitated/defibrillated patients with out-of-hospital cardiac arrest (OHCA) due to acute ischemia and (4) STEMI/NSTEMI patients with a high thrombotic burden. Primary endpoint is 48 h Net Adverse Clinical Events (NACE), which is a composite endpoint of all-cause death, recurrent myocardial infarction (MI), target vessel revascularization (TVR), stroke, stent thrombosis (ST) and BARC 2-3-5 bleeding.

**Summary:**

The Dutch Cangrelor Registry will assess the feasibility and safety of cangrelor in patients with suboptimal P2Y_12_ inhibition undergoing (primary) PCI in the setting of acute coronary syndrome (ACS) and stable coronary artery disease (CAD) in the Netherlands.

## Introduction

Oral dual antiplatelet therapy (DAPT), consisting of aspirin and a P2Y_12_-receptor inhibitor, is the cornerstone of treatment in patients with an acute coronary syndrome (ACS) and stable coronary artery disease (CAD) undergoing (primary) percutaneous coronary intervention (PCI) to prevent adverse ischemic complications [1]. Clopidogrel as well as the stronger P2Y_12_ inhibitors ticagrelor and prasugrel are the recommended and most commonly used oral P2Y_12_ inhibitors in the Netherlands [2, 3]. Ticagrelor and prasugrel provide faster, more potent, and more consistent P2Y_12_ inhibition compared with clopidogrel, and have been associated with a lower rate of adverse ischemic events [4, 5]. Despite the advances of these stronger P2Y_12_ antagonists, all oral P2Y_12_ inhibitors pose a relatively slow onset of inhibition in the first hours after intake [6, 7]. In addition, P2Y_12_ inhibition by oral agents may be insufficient due to ST-segment Elevation Myocardial Infarction (STEMI)-induced selective shunting of blood to vital organs, which decreases gastro-intestinal perfusion with impaired absorption of the oral P2Y_12_ inhibitors as a result. The bioavailability of the oral agents is further reduced in the presence of vomiting of the loading dose or therapeutic hypothermia [8, 9]. Furthermore, concomitant administration of morphine or fentanyl for pain relief and an oral P2Y_12_ inhibitor, might lead to significantly reduced or delayed absorption of the latter [10–12]. Therefore, intravenous administration of a P2Y_12_ inhibitor might be beneficial to overcome the limitations of oral P2Y_12_ inhibitors and might bridge the gap to optimal platelet inhibition by oral P2Y_12_ inhibitors only.

Cangrelor, an intravenous adenosine diphosphate (ADP)-receptor antagonist, is a potent and rapidly acting P2Y_12_ inhibitor with fast reversible effects. Its clinical use has been studied in three large-scale trials in the setting of ACS, referred to as the CHAMPION (Cangrelor versus Standard Therapy to Achieve Optimal Management of Platelet Inhibition) trials [13–15]. In the CHAMPION-PHOENIX trial with 11,145 patients undergoing elective or urgent revascularization, cangrelor showed a significant reduction of composite endpoint of all-cause mortality, myocardial infarction (MI), ischemia-driven revascularization, or stent thrombosis (ST) at 48 h (4.7% vs. 5.9%; *P* = 0.005), without a significant increase in severe bleeding (0.16% vs. 0.11%; *P* = 0.44), compared with clopidogrel without pre-treatment with cangrelor [16]. This was mainly driven by less MIs in the cangrelor group and was consistent among patients presenting with STEMI, non ST-segment elevation myocardial infarction (NSTEMI), and those presenting with stable CAD. The CHAMPION-PCI trial, in which cangrelor was compared with pre-interventional administered clopidogrel, also showed no significant difference in GUSTO-defined major bleeding between the two agents [17]. However, it failed to show superiority of the addition of cangrelor to clopidogrel with regard to ischemic events in patients with ACS and stable CAD compared to clopidogrel only. The same applied for the CHAMPION-PLATFORM trial, in which cangrelor was compared with post-interventional administered clopidogrel [18]. It showed only a significant reduction of death by all causes and ST in the subgroup of NSTEMI. Though, a pooled analysis of all 3 CHAMPION trials showed a reduction in peri-procedural ischemic complications in patients undergoing PCI at the expense of a small increase in mild bleeding [19].

Limited data is available about the stronger P2Y_12_ inhibitors, ticagrelor and prasugrel, in combination with cangrelor [20–22]. The CANTIC (Platelet Inhibition with Cangrelor and Crushed Ticagrelor in Patients with STEMI Undergoing Primary Percutaneous Coronary Intervention) trial evaluated the additional effect of cangrelor with crushed ticagrelor in STEMI patients undergoing primary PCI and showed more potent platelet inhibitory effects with reduction of platelet reactivity as early as 5 min after cangrelor infusion compared with crushed ticagrelor alone [23]. The FABULOUS FASTER trial was the first randomized study to directly compare the pharmacodynamic effects of cangrelor with prasugrel and tirofiban. Tirofiban, another intravenous antiplatelet drug which belongs to the class of Glycoprotein IIb/IIIa inhibitors (GPIs), yielded superior inhibition of platelet aggregation (IPA) over cangrelor at 30 min after infusion. However, cangrelor and tirofiban were both superior to chewed prasugrel [41].

Since various studies have been conducted with cangrelor, there is little real-life data on the use of cangrelor in daily clinical practice. Hence, we propose a registry in the Netherlands, studying the feasibility and safety of cangrelor in high thrombotic risk patients with suboptimal P2Y_12_ inhibition who undergo (primary) PCI.

## Methods

### Study design

The Cangrelor Registry is an open-label, prospective, multicenter, single-arm study which aims to assess the feasibility and safety of cangrelor in: (1) P2Y_12_ naive patients with ad-hoc PCI, (2) patients with STEMI/NSTEMI with suboptimal P2Y_12_ inhibition including (3) stable resuscitated/defibrillated patients with out-of-hospital cardiac arrest (OHCA) due to acute ischemia, and (4) STEMI/NSTEMI patients with a high thrombotic burden.

The study will be conducted in 8 centers in the Netherlands: Isala Hospital (Zwolle), St. Antonius Hospital (Nieuwegein), Zuyderland Medical Center (Heerlen), Catharina Hospital (Eindhoven), Venlo VieCuri Medical Center (Venlo), OLVG (Amsterdam), University of Medical Center Groningen (Groningen), and Medical Center Leeuwarden (Leeuwarden). The study complies with the Declaration of Helsinki and is approved by the institutional review board (local medical ethics committee).

All patients, except resuscitated patients from cardiac arrest, will provide verbal informed consent for the participation prior to coronary angiography. Written informed consent will be obtained after the PCI. In the case of OHCA patients, their legal representative will be informed by the interventional cardiologist and will sign the informed consent on behalf of the patient.

### Study protocol, patient enrollment and follow up

On admission, the medical team will perform physical examination, register vital parameters, electrocardiogram and laboratory assessments, and will set the diagnosis. All consecutive patients who fulfill the inclusion and exclusion criteria will be eligible for enrollment. Table 1 summarizes the inclusion and exclusion criteria. The indication for cangrelor and actual enrollment will be at the discretion of the treating physician. The timing of administration of cangrelor during the procedure is pre-specified: cangrelor bolus and infusion will be administered after initial coronary angiography, but before the start of PCI. PCI will be performed according to standard procedures. During the PCI, the initial Thrombolysis In Myocardial Infarction (TIMI) flow, TIMI flow post-PCI and myocardial blush grade (MBG) will be noted.

**Table 1:** Overview of inclusion and exclusion criteria

Inclusion criteria
Age > 18 years
Able to give informed consent
One of the following criteria: Patients naive for P2Y_12_ inhibition undergoing ad-hoc PCI Patients with STEMI/NSTEMI loaded with oral P2Y_12_ inhibitors though platelet inhibition still insufficient (< 2 h after oral loading dose) according to operator Patients with STEMI/NSTEMI who vomited after P2Y_12_ loading dose Patients with OHCA, based on VF/VT due to acute ischemia as the underlying cause, with Return of Spontaneous Circulation (ROSC) who have successfully been defibrillated and/or resuscitated with stable hemodynamics STEMI/NSTEMI patients loaded with oral P2Y_12_ inhibitors with large thrombus burden (TBG 4 or 5) on initial coronary angiography and undergoing (primary) PCI with expected insufficient P2Y_12_ inhibition

Exclusion criteria
Patients on current/chronic treatment with P2Y_12_ inhibitors
Patients (pre)treated with GPIs
Patients with recent major bleeding complications or contraindication to DAPT: Hypersensitivity or allergy to and known contraindication to aspirin, clopidogrel, ticagrelor, prasugrel, or cangrelor History of major clinical bleeding or known coagulopathy Active bleeding History of intracerebral mass, aneurysm, arteriovenous malformation, or hemorrhagic stroke Known severe liver dysfunction
Patients that have received any organ transplant or await any organ transplant
Patients undergoing dialysis
Pregnant or lactating female
Patients currently participating in another investigational drug or drug-coated device study

PCI, percutaneous coronary intervention; OHCA, Out of Hospital Cardiac Arrest; VF, ventricular fibrillation; VT, ventricular tachycardia; TBG, thrombus burden grade; GPI, Glycoprotein IIb/IIIa inhibitor; DAPT, Dual antiplatelet therapy

After PCI, adverse events will be assessed at 48 h and 30 days post-PCI. At 48 h after PCI, the electronic medical records will be consulted to assess the occurrence of any adverse events. Since most resuscitated patients will still be hospitalized at 48 h, the electronical medical records will be sufficient to determine the adverse events in these patients. If the medical records are insufficient, then a telephone evaluation with the patient will be conducted. The 30 days follow up will be performed by a telephone interview.

### Pharmacological treatment

STEMI patients will receive concomitant medication according to European Guidelines including intravenous acetylsalicylic acid 500 mg, an oral loading dose of 180 mg of ticagrelor, 600 mg of clopidogrel or 60 mg of prasugrel, and an intravenous bolus of 5000 units of heparin, by the paramedical team (i.e. ambulance) or the medical team before the primary PCI. NSTEMI patients will receive intravenous acetylsalicylic acid 500 mg, an oral loading dose of 180 mg of ticagrelor, 600 mg of clopidogrel or 60 mg of prasugrel, and subcutaneous fondaparinux 1.5 mg or 2.5 mg till the coronary angiography.

STEMI/NSTEMI patients with expected suboptimal P2Y_12_ inhibition and/or with high thrombus burden will receive cangrelor bolus and infusion before wire passage of the culprit lesion.

Patients with stable CAD awaiting coronary angiography will be on aspirin 80 mg per day. After PCI, they will receive an oral loading dose of ticagrelor, clopidogrel or prasugrel.

### Cangrelor

Cangrelor is a currently approved P2Y_12_-purinoreceptor antagonist, which is available for intravenous use in clinical care. It is a potent inhibitor of ADP-induced aggregation of human platelets, which acts directly at P2Y_12_-receptors and does not require conversion in the liver to an active metabolite, with rapid onset after intravenous infusion. Furthermore, plasma concentrations of cangrelor are unaffected by renal or hepatic impairment [42]. The short half-life of 3–6 min of cangrelor results in a rapid offset of antiplatelet effect and the effect on the bleeding time within 20 min after discontinuation of the infusion. Its advantages over an oral P2Y_12_ inhibitor include more potent and rapid P2Y_12_ inhibition and potentially lower bleeding risk.

Cangrelor is titrated on weight. First, a bolus of 30 µg/kg will be administered, then the infusion will be started based on 4 µg/kg/min. When transitioning to oral P2Y_12_ inhibitors, the loading dose of ticagrelor can be given at any time during the cangrelor infusion or immediately after discontinuation of the infusion [23]. The loading dose of clopidogrel or prasugrel will be given immediately after the discontinuation of the cangrelor infusion, because of drug-drug interaction [24]. Patients who vomited after an oral loading dose of ticagrelor, clopidogrel or prasugrel will receive another loading dose before the coronary angiography or after the revascularization if they had received ticagrelor, or after the end of cangrelor infusion if they had received clopidogrel or prasugrel. Patients naive for P2Y_12_ inhibition with the indication for ad-hoc PCI, will receive the oral loading dose of clopidogrel and prasugrel after the intervention and the oral loading dose of ticagrelor often earlier. Type and duration of chronic oral P2Y_12_ inhibition with clopidogrel, ticagrelor or prasugrel will be at discretion of the treating physician.

### Endpoints

The primary endpoint is a composite efficacy and safety endpoint of all-cause mortality (including cardiac death), (recurrent) MI, target vessel revascularization (TVR), stroke, probable or definite ST and, bleeding (according to Bleeding Academic Research Consortium [BARC] type 2-3-5) at 48 h after (primary) PCI [25–27].

The secondary endpoint is the composite endpoint of all-cause mortality (including cardiac death), recurrent MI, target vessel revascularization (TVR), stroke, definite or probable ST, and bleeding (BARC type 2-3-5) at 30 days after (primary) PCI. Moreover, all individual endpoints will be assessed.

### Sample size and statistical considerations

The current enrollment target is 250 patients across the 8 centers in the Netherlands. This is an observational study designed to provide descriptive summary information and therefore a comparison group is absent. As such, no formal power calculation has been performed. This size is considered as a good balance between feasibility to provide clinically meaningful information within the projected time period of 1 year.

Descriptive statistics will be performed for baseline characteristics and both primary and secondary endpoints. Continuous variables will be expressed as means with standard deviation, or as medians with 25th and 75th percentiles. Categorical variables will be expressed as frequencies with percentages.

## Expected results

The Dutch Cangrelor Registry will explore the feasibility and safety of cangrelor in P2Y_12_ naive patients with ad-hoc PCI, in STEMI/NSTEMI patients with suboptimal P2Y_12_ inhibition including stable resuscitated/defibrillated patients with OHCA due to acute ischemia as the underlying cause, and/or in STEMI/NSTEMI patients with a high thrombotic burden, who all undergo (primary) PCI.

## Discussion

### The importance of optimal P2Y_12_ inhibition

(Primary) PCI with implantation of best in class drug-eluting stents (DESs) is the recommended treatment for flow restoration of an infarct-related artery in the setting of ACS or in patients with stable CAD with significant coronary artery stenosis [28]. Subsequently, intensive anti-thrombotic therapy with optimal P2Y_12_ inhibition through DAPT is crucial to prevent adverse ischemic complications with ST as most fierce complication.

### Optimal P2Y_12_ inhibition in whom?

In particular, ACS patients with high thrombus burden (TIMI Thrombus Grade 4 or 5) with suboptimal P2Y_12_ inhibition are associated with a higher incidence of major adverse cardiovascular events (MACEs) [29–34]. Another example are the P2Y_12_ naive patients with stable CAD, who are considered for ad-hoc PCI. They are not able to receive immediate stenting due to the absence of P2Y_12_ inhibition and are once more exposed to contrast agents at the time of PCI. In these contexts, the use of a higher loading dose of oral P2Y_12_ inhibitors has been proven ineffective to facilitate potent P2Y_12_ inhibition, as well as crushing tablets which gives only approximately 2 h of gain of early P2Y_12_ inhibition compared with the oral agents [35, 36]. As a result, an intravenous P2Y_12_ inhibitor may bridge this gap to standard care with oral P2Y_12_ inhibitors and avoids postponing PCI. This also applies for defibrillated/resuscitated patients with OHCA as a result of acute ischemia. The survivors of OHCA, whether conscious or comatose after ROSC, are not able to take oral P2Y_12_ inhibitors. Due to the need for nasogastric or orogastric tube insertion, there is an important delay until optimal P2Y_12_ inhibition is realized. Moreover, there are other factors that influence the pharmacokinetics of oral P2Y_12_ inhibitors, especially in comatose patients of OHCA, such as mild therapeutic hypothermia (MTH), gastroparesis, hypoperfusion of gastrointestinal tract and platelet hyperreactivity as a result of systemic inflammatory response syndrome (SIRS) [37]. These characteristics put this category of patients at risk of acute and subacute ST leading to increased morbidity and mortality. This underlines the continued unmet clinical need for a more potent, rapid-acting, and safe antiplatelet intravenous agent in patients undergoing PCI.

### Optimizing P2Y_12_ inhibition

Cangrelor can be considered in P2Y_12_ naive patients or in ACS patients with suboptimal P2Y_12_ inhibition undergoing (primary) PCI. Cangrelor may also be useful in other clinical settings. For example in the context of coronary artery bypass grafting (CABG), pretreatment with oral P2Y_12_ inhibitors may delay revascularization and may unnecessarily increase the risk of bleeding [38]. In this setting, cangrelor may represent a valuable option due to the rapid offset of P2Y_12_ inhibition after discontinuation.

Another group of intravenous antiplatelet drugs is represented by the glycoprotein IIb/IIIa inhibitors (GPIs), which provide rapid and effective antiplatelet inhibition by competing with von Willebrand factor and fibrinogen for GPIIb/IIIa receptor binding. In comparison with cangrelor, GPIs inhibit platelet reaction to all agonists, which induces nearly complete IPA. However, despite their ischemic benefits the activity of GPIs persist for hours after discontinuation which might lead to higher risk of bleeding, especially when surgery is needed shortly after coronary angiography or PCI [39, 40].

## Strengths and limitations

Our study’s strength is that it represents patients in the daily clinical practice with wide inclusion criteria in a multicentre design. However, the registry has some limitations, as it is an open label trial with a small cohort of patients without a control group. Secondly, given the study’s observational nature, the results may be influenced by (unmeasured) confounding factors. Finally, worldwide differences in health care systems and the availability of cangrelor is a point of discussion.

## Conclusion

In the Dutch Cangrelor Registry, we aim to investigate the feasibility and safety of cangrelor in 250 patients with suboptimal P2Y_12_ inhibition undergoing (primary) PCI in daily clinical practice in 8 large interventional hospitals in the Netherlands.

## Data Availability

All data that will generated and analyzed during this study will be published in the main article.

## Abbreviations

ACS: Acute Coronary Syndrome
ADP: Adenosine Diphosphate
AE: Adverse Event
AP: Angina pectoris
BARC: Bleeding Academic Research Consortium
CAD: Coronary artery disease
CABG: Coronary Artery Bypass Grafting
DAPT: Dual antiplatelet therapy
GPI: Glycoprotein IIb/IIIa inhibitor
IPA: Inhibition of platelet aggregation
MI: Myocardial infarction
MTH: Mild therapeutic hypothermia
NACE: Net Adverse Clinical Events
NSTEMI: Non ST-segment Elevation Myocardial Infarction
OHCA: Out of Hospital Cardiac Arrest
PCI: Percutaneous coronary intervention
ROSC: Return of Spontaneous Circulation
SAE: Severe Adverse Event
SIRS: Systemic inflammatory response syndrome
ST: Stent thrombosis
STEMI: ST-segment Elevation Myocardial Infarction
TIMI: Thrombolysis in Myocardial Infarction
